# T-tube enterostomy in the management of apple-peel atresia: A case series from a single center

**DOI:** 10.3389/fped.2022.1003508

**Published:** 2022-11-09

**Authors:** Hayssam Rashwan, Mostafa Kotb

**Affiliations:** ^1^ Pediatric Surgery, Alexandria Faculty of Medicine, Alexandria, Egypt; ^2^ Pediatric Surgery, Alexandria Faculty of Medicine, Alexandria, Egypt

**Keywords:** apple-peel atresia, intestinal atresia, neonates, Stamm technique, T-tube

## Abstract

**Background and objective:**

Although complex atresias, such as apple-peel and multiple atresias, comprise a smaller percentage, they are usually associated with a higher incidence of postoperative complications and mortality rate. Contrary to simple atresias where the surgical technique of choice usually entails bowel resection and anastomosis with or without enteroplasty, managing apple-peel atresia remains more sophisticated. Decompressive and functionalizing stomas are sometimes mandatory to overcome problems such as increased wall thickness and the wide disparity among the anastomotic ends. Few reports discussed using tube enterostomy in the management of apple-peel atresia; nonetheless, no previous prospective studies were conducted to discuss its efficacy on a larger population. In this study, we are describing our experience using this technique on 12 patients suffering from apple-peel atresia in our center.

**Methods:**

A prospective study was conducted from June 2015 to May 2020, where all children who were found to have apple-peel atresia were included in the study. T-tube was placed through an enterotomy through the dilated proximal bowel, around 10 cm before the anastomotic line, and was kept in place using a double suture (Stamm technique) before closing the anterior face of the anastomosis. The short distal limb of the T-tube was oriented toward the anastomotic line, while the long proximal limb was directed proximally. After finishing the anastomosis, the T-tube was delivered outside the abdominal wall, anchoring the enterostomy along with the proximal dilated jejunum against the anterior abdominal wall.

**Results:**

A total of 12 cases were encountered throughout the period of study. The mean age at operation was 4 days and the mean birth weight was 2700 g. The mean time for starting oral feeding postoperatively and T-tube removal was 8 and 10.5 days, respectively. Cases were discharged after a mean of 22 days. As regards morbidity and mortality, a single case developed skin excoriations at the site of tube insertion and was managed conservatively using topical ointments and another case died from overwhelming sepsis 3 days after the operation.

**Conclusion:**

T-tube enteroplasty is a safe and feasible option in the surgical management of apple-peel atresia. The main strength of our study is its prospective nature and that it includes apple-peel atresia cases only. However, the main limitation is that a larger sample is needed.

## Introduction

Small bowel atresia is a common cause of neonatal intestinal obstruction. Fortunately, the majority of intestinal atresias are of the simple type, which is characterized by a favorable anatomy for re-establishing intestinal continuity. On the other hand, complex atresias, such as type IIIb atresia (apple-peel) and type IV atresia (multiple atresias), comprise a smaller percentage and are associated with a higher incidence of postoperative complications and mortality rate (1, 2). These babies are usually premature and of low birth weight. Additionally, they may have associated anomalies such as malrotation and may develop short bowel syndrome (SBS) with may increase morbidity and mortality rates (3). Since this complex subtype of atresia was initially described, several authors have reported their experiences in the management of apple-peel atresia. Roughly, most of these cases were managed by primary end-to-end linear anastomosis (4), while others resort to double enterostomies, or partial anastomoses, whether end-to-side or side-to-end (5–7). Marked improvement in survival was partially attributable to enhanced surgical technique, and mainly due to improved perioperative management and especially total parenteral nutrition (TPN). Since prolonged TPN is associated with considerable morbidity, surgical procedures should focus on reducing the number of surgical procedures, and therefore, the length of TPN (8). Few reports discussed the use of tube enterostomy in the management of apple-peel atresia aiming to decrease the number of surgical procedures, avoid resorting to bowel resection with resulting SBS and early commencing oral feeds to reduce the length of TPN (9). Nevertheless, no previous prospective studies were conducted to discuss its efficacy on a larger population. In this study, we are describing our experience using this technique on 12 patients suffering from apple-peel atresia in our center.

## Methods

This was a prospective study that was conducted from June 2015 to May 2020. After approval of the ethics committee of Alexandria Faculty of Medicine, parents and legal guardians were informed well about the procedure and informed written consent was collected before carrying out the procedure. Once admitted, the patients’ demographic data were collected followed by a thorough history and physical examination. An erect abdominal x-ray was performed to confirm the diagnosis of jejunal atresia. All patients proved to have apple-peel jejunal atresia intraoperatively were included in the study.

**Figure 1:**
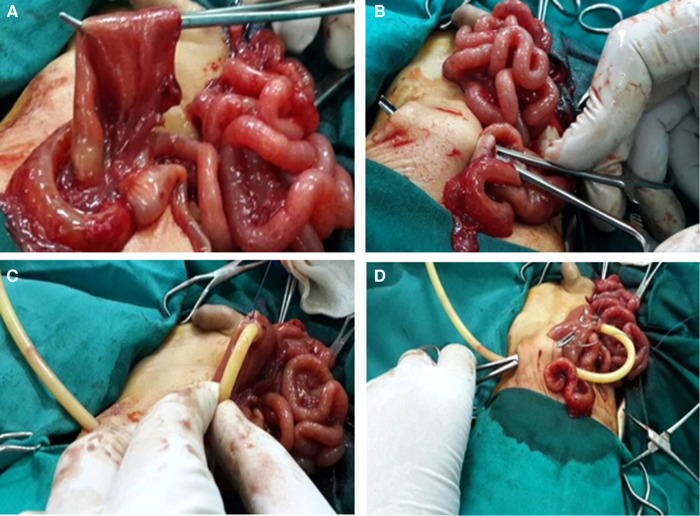
(**A**) Enterotomy performed through the proximal dilated jejunum. (**B**) Incision through the anterior abdominal wall to provide route for T-tube. (**C**) T-tube secured in place by purse-string sutures. (**D**) T-tube exteriorized through the abdominal wall fixing the enterostomy and ensuring the proximal dilated jejunum against the anterior abdominal wall.

An exploratory right transverse muscle-cutting incision was performed in all cases. Once a diagnosis of apple-peel atresia was confirmed intraoperatively, an extramucosal end-to-end intestinal anastomosis with 5/0 vicryl suture was performed. Before closing the anterior face of the anastomosis, the T-tube was placed through an enterotomy through the dilated proximal bowel, around 10 cm before the anastomotic line, and was kept in place using double suture (Stamm technique). The short distal limb of the T-tube was oriented past the anastomotic line, i.e., transanastomotic, while the long proximal limb was directed proximally. After finishing the anastomosis, the T-tube was delivered outside the abdominal wall, anchoring the enterostomy along with the proximal dilated jejunum against the anterior abdominal wall (Figure 1).

**Figure 2:**
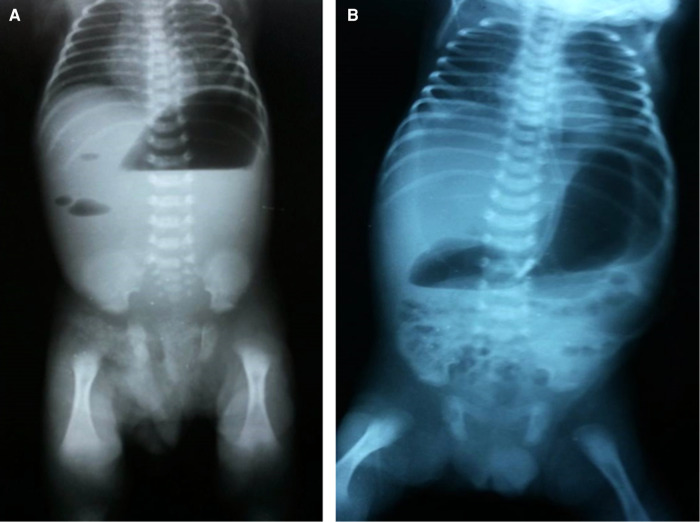
Erect x-ray of the abdomen. (**A**) At admission. (**B**) Three days postoperatively.

Postoperatively, the nasogastric tube was kept in place, parenteral nutrition was initiated until bowel recovery. The onset of oral feeding was gradual depending on the resumption of the intestinal function including low-volume clear gastric residue and active defecation. Removal of the T-tube was performed when the contrast study ensured free distal flow. Patients were discharged if the following criteria were met: adequate wound healing, regular oral feeding intake and bowel movement (Figure 2). Patients were followed up in the clinic after 1, 3, 6, 12, and 24 months following the discharge where the wound complications, bowel habits and developmental milestones were monitored thoroughly during the visit.

## Results

A total of 12 cases were encountered throughout the period of study. The mean age of included neonates at operation was 4 days (range of 3–11 days), while the mean birth weight was 2700 g (range of 2200–3600 g). The mean time for starting oral feeding postoperatively was 8 days (range 6–17 days) and the mean time for T-tube removal was 10.5 days (range 8–18 days). Spontaneous closure was attained in all cases, i.e., none of the patients developed enterocutaneous fistula or required any additional surgeries. Cases were discharged after a mean of 22 days (range 19–34 days). As regards morbidity and mortality, a single case developed skin excoriations at the site of tube insertion and was managed conservatively using topical ointments and another case died from overwhelming sepsis 3 days after the operation. The mean follow-up period was 7.5 months (6–24 months).

## Discussion

Apple-peel atresia remains a challenging subtype among other cases of intestinal atresia and an important cause of SBS (10). Operative intervention for surgical repair of such complex anatomy entails resection and anastomosis with or without enteroplasty. Recently, Han et al. (11) studied mesopexy after primary anastomosis by retrograde small bowel mesentery fixation starting from the ileocecal region and proceeding around the enlarged blind pouch and reported mortality of 3 cases only from 42 patients. The other option for apple-peel atresia treatment is performing temporary stoma, which is sometimes indicated in cases with questionable bowel viability, significant peritonitis, or caliber discrepancies between both ends.

Since there is no standard treatment, many authors proposed the use of intubated enterostomies differently; nonetheless, most of these reports were case reports or case series. Federici et al. used intraluminal silicone tubes in five type IIIb atresia cases, with successful results (12). Another method suggested by Elhalaby is to perform a primary anastomosis with a tube proximally directed through the cecum into the small intestine in order to preserve the maximal length of the intestine in a preterm type IIIa jejunal atresia newborn. This tube served as a stent for the anastomosis and made it more feasible for the proximal segment's contents to decompress (13). Two case reports were published describing the use of Foley’s catheter and T-tube, using transanastomotic feeding tubes in both cases (9, 14). Al-Zaiem et al. performed a retrospective study on the use of a T-tube in neonatal gastrointestinal surgery over 62 neonates including 34 jejunoileal atresias; however, no specifications on the number of apple-peel cases encountered in his study (15).

T-tube enterostomy offers many advantages when compared with other techniques used in treating proximal jejunal atresia such as resection of the dilated part, tapering, and/or stoma. When compared with primary end-to-end linear anastomosis, T-tube obviates the need to remove the dilated ectatic hypoperistaltic segment and offers the chance for early commencement of oral feeding *via* a transanastomotic distal limb. This reduces the length of TPN and hospital stay. In a study by Xu et al. (16), the length of TPN administration and hospital stay were around 37 days, compared with 8 and 22 days, respectively, in our study. This reduces the costs and risk of sepsis with avoidance of many TPN-related complications such as line sepsis, metabolic, and hepatic implications. In addition, by avoiding the resection of the dilated part, the neonate could benefit from the preservation of a higher absorptive surface; hence, reducing the risk of developing SBS. It can be also suitable when the dilated bowel is within very close proximity to the duodenojejunal flexure and hence very critical to be resected. The risk of leakage is lower than tapering as the latter has an additional anastomotic line, which adds to the risk of leakage (9).

T-tube enterostomy, when compared with a stoma, prevents stoma-related complications like fluid and electrolyte imbalance, the need for prolonged TPN, as well as skin excoriations, and avoids the need for a second operation because the T-tube can be removed safely as a bedside maneuver in the NICU with spontaneous closure of the enterocutaneous fistula. T-tube enteroplasty provided two more benefits. The dilated loop is first fixed against the abdominal wall by the proximal long limb of the T-tube, which stabilizes the anastomotic opening and promotes antegrade peristalsis. Lastly, it facilitates contrast studies of the bowel should anastomotic leakage or stenosis were suspected (9).

To sum up, from our prospective, T-tube enteroplasty is a safe and feasible option in the surgical management of apple-peel atresia. The main strength of our study is its prospective nature and that it includes apple-peel atresia cases only. However, the main limitation is that a larger sample is needed as well as a longer period of follow-up.

## Data Availability

The raw data supporting the conclusions of this article will be made available by the authors, without undue reservation.
